# Clinical significance of long non-coding RNA *HOTTIP* in early-stage non-small-cell lung cancer

**DOI:** 10.1186/s12890-019-0816-8

**Published:** 2019-02-28

**Authors:** Alfons Navarro, Jorge Moises, Sandra Santasusagna, Ramon M. Marrades, Nuria Viñolas, Joan J. Castellano, Jordi Canals, Carmen Muñoz, José Ramírez, Laureano Molins, Mariano Monzo

**Affiliations:** 1 Molecular Oncology and Embryology Laboratory, Human Anatomy Unit, School of Medicine, University of Barcelona, IDIBAPS, Casanova 143, 08036 Barcelona, Spain; 2 Department of Pneumology, Institut Clínic de Respiratori (ICR), Hospital Clínic de Barcelona, University of Barcelona, IDIBAPS, CIBER de Enfermedades Respiratorias (CIBERES), Barcelona, Spain; 3 Department of Medical Oncology, Institut Clínic Malalties Hemato-Oncològiques (ICMHO), Hospital Clínic de Barcelona, University of Barcelona, IDIBAPS, Barcelona, Spain; 4 Department of Pathology, Centro de Diagnóstico Biomédico (CDB), Hospital Clínic de Barcelona, University of Barcelona, IDIBAPS, CIBERES, Barcelona, Spain; 5 Department of Thoracic Surgery, Institut Clínic de Respiratori (ICR), Hospital Clínic de Barcelona, University of Barcelona, Barcelona, Spain

**Keywords:** HOTTIP, NSCLC, Early-stage, Overall survival, Lung cancer, lncRNAs

## Abstract

**Background:**

*HOTTIP*, a long non-coding RNA located in the *HOXA* cluster, plays a role in the patterning of tissues with mesodermal components, including the lung. Overexpression of *HOXA* genes, including *HOTTIP*, has been associated with a more aggressive phenotype in several cancers. However, the prognostic impact of *HOTTIP* has not yet been explored in non-small-cell lung cancer (NSCLC). We have correlated HOTTIP expression with time to relapse (TTR) and overall survival (OS) in early-stage NSCLC patients.

**Methods:**

Ninety-nine early-stage NSCLC patients who underwent surgical resection in our center from June 2007 to November 2013 were included in the study. Mean age was 66; 77.8% were males; 73.7% had stage I disease; and 55.5% had adenocarcinoma. A validation data set comprised stage I-II patients from The Cancer Genome Atlas (TCGA) Research Network.

**Results:**

*HOTTIP* was expressed in all tumor samples and was overexpressed in squamous cell carcinoma (*p* = 0.007) and in smokers (*p* = 0.018). Patients with high levels of *HOTTIP* had shorter TTR (78.3 vs 58 months; *p* = 0.048) and shorter OS (81.2 vs 61 months; *p* = 0.023) than those with low levels. In the multivariate analysis, *HOTTIP* emerged as an independent prognostic marker for TTR (OR: 2.05, 95%CI: 1–4.2; *p* = 0.05), and for OS (OR: 2.31, 95%CI: 1.04–5.1; *p* = 0.04). *HOTTIP* was validated as a prognostic marker for OS in the TCGA adenocarcinoma cohort (*p* = 0.025). Moreover, we identified a 1203-mRNA and a 61-miRNA signature that correlated with *HOTTIP* expression.

**Conclusions:**

The lncRNA *HOTTIP* can be considered a prognostic biomarker in early-stage NSCLC.

**Electronic supplementary material:**

The online version of this article (10.1186/s12890-019-0816-8) contains supplementary material, which is available to authorized users.

## Background

According to the American Cancer Society, in 2018, lung cancer will be the second most frequent cancer in the United States of America in both males and females (14 and 13%, respectively, of all cancers) and the first leading cause of death by cancer (26 and 25%, respectively) [1]. Non-small-cell lung cancer (NSCLC), the most frequent subtype, accounts for 85% of all lung cancers. Despite years of research, the prognosis for patients with NSCLC remains dismal, with a 5-year relative survival rate of 18% for all stages combined (www.cancer.net). In the 30% of patients that debut with early-stage disease (stage I-II), the cornerstone of treatment is the surgical removal of the tumor. Moreover, in stage IB disease with a primary tumor > 4 cm and in stage II disease, adjuvant chemotherapy (usually cisplatin-vinorelbine) has proven to be beneficial, with a 4–5% absolute survival improvement at five years [2]. A large study including 1294 consecutive early-stage NSCLC patients who underwent surgery showed that after a median follow up of 35 months, 20% of patients had relapsed and 7% were diagnosed with a second primary lung cancer [3]. These data highlight the need to further investigate this disease and consolidate useful prognostic markers.

Up to 70% of our genome is transcribed into non-coding RNAs (ncRNAs) that do not serve as templates for proteins. These ncRNAs are subdivided into two major groups: small ncRNAs (< 200 nt) and long ncRNAs (lncRNAs) (> 200 nt) [4]. Although small ncRNAs, especially microRNAs (miRNAs), have been the most extensively studied [5], lncRNAs have recently emerged as worthy biomarkers, since their expression is highly cell type- and tissue-specific [6]. In NSCLC, several lncRNAs are involved in the carcinogenesis process, some of which have been associated with patient survival [7–9].

The *HOX* family genes are known transcription factors with a key role in embryogenesis and carcinogenesis [10, 11]. Their expression is dysregulated in several cancers, including NSCLC [11–14]. In humans, *HOX* genes are organized into four clusters (A, B, C, and D), which are located on different chromosomes [15]. Interestingly, several lncRNAs associated with *HOX* genomic regions can participate in the regulation of *HOX* genes and collaborate in their functions [16]. *HOTTIP*, also known as HOXA distal transcript antisense RNA, is an antisense lncRNA located in the *HOXA* cluster that coordinates the activation of several 5′ *HOXA* genes in vivo [17]. It is overexpressed in several cancers [18–20], including NSCLC, where its overexpression in vitro has been associated with increased proliferation and invasion of lung cancer cells through transcriptional regulation of *HOXA13* [21]. Moreover, *HOTTIP* overexpression has been associated with worse outcome in several tumors, including hepatocellular carcinoma [22], tongue squamous cell carcinoma [18], colorectal cancer [20], osteosarcoma [23], breast cancer [24], gastric cancer [19], and even small-cell lung cancer [25]. To date, however, the prognostic impact of *HOTTIP* expression levels in NSCLC has not been explored [26, 27].

In the present study, we have analyzed *HOTTIP* expression in a cohort of 99 patients with early-stage NSCLC who underwent surgical resection in our center and have correlated *HOTTIP* expression levels with overall survival (OS) and time to relapse (TTR).

## Methods

### Patients

A total of 99 early-stage NSCLC patients who underwent complete surgical resection in Hospital Clínic (Barcelona) from May 2007 to October 2013 were included in the study. Prospectively collected tumor tissues were stored in RNALater® (Ambion) at − 80 °C until processing. Clinical data were recorded on admission: age, gender, smoking history, Eastern Cooperative Oncology Group (ECOG) performance status (PS), preoperative pulmonary function tests (PFT) and chronic obstructive pulmonary disease (COPD). COPD was defined as when forced expiratory volume in 1 s (FEV1) to forced vital capacity (FVC) ratio is below a fixed cutoff (< 70%). Type of surgical resection and pathological findings were also recorded, including tumor characteristics and the presence of emphysema (defined histopathologically in the resected non-tumoral tissue). All patient samples were studied by Sanger sequencing for mutations in TP53 (exons 5–8) and KRAS (codon 12–13), and only adenocarcinoma patients samples were studied for EGFR mutations (exons 19–21). The following primers were used: TP53 exon 5 Forward (F) 5′- GTTTCTTTGCTGCCGTCTTC-3′, TP53 exon 5 Reverse (R) 5′-GAGCAATCAGTGAGGAATCAGA-3′; TP53 exon 6 F 5′-AGAGACGACAGGGCTGGTT-3′, TP53 exon 6 R 5′-CTTAACCCCTCCTCCCAGAG-3′; TP53 exon 7 F 5′- TTGCCACAGGTCTCCCCAA-3′, TP53 exon 7 R 5′-AGGGGTCAGAGGCAAGCAGA-3′; and TP53 exon 8 F 5′-GGGACAGGTAGGACCTGATTT-3′, TP53 exon 8 R 5′-TAACTGCACCCTTGGTCTCC-3′; KRAS F 5′-TTAACCTTATGTGTGACATGTT-3′, KRAS R 5′-AGAATGGTCCTGCACCAGTAA-3′; EGFR exon 18 F 5′- GCATGGTGAGGGCTGAGGT-3′, EGFR exon 18 R 5′-TGCAAGGACTCTGGGCTCC-3′,EGFR exon 19 F 5′-TGCATCGCTGGTAACATCCA-3′, EGFR exon 19 R 5′-GAAAAGGTGGGCCTGAGGTT-3′, EGFR exon 20 F 5′-TCCTTCTGGCCACCATGC-3′, EGFR exon 20 R 5′-TGGCTCCTTATCTCCCCTCC-3′, EGFR exon 21 F 5′-ATGCAGAGCTTCTTCCCATGA-3′, EGFR exon 21 R 5′-CAGGAAAATGCTGGCTGACC-3′.

All patients signed the written consent in accordance with the Declaration of Helsinki to use their samples in the present research. The Clinical Research Ethics Committee of the Hospital Clínic de Barcelona approved the study.

NSCLC patients from The Cancer Genome Atlas (TCGA) Research Network (RNAseq data; https://cancergenome.nih.gov) were used as a validation data set. Two cohorts were used: the Lung Adenocarcinoma (TCGA-LUAD) and the Lung Squamous Cell Carcinoma (TCGA-LUSC). The patient selection criteria were: stage I-II, Caucasian, minimum OS of 35 days, and available RNAseq data. Using these selection criteria, the validation cohorts included 91 adenocarcinomas from the TCGA-LUAD and 59 squamous cell carcinomas from the TCGA-LUSC cohort. The analysis was performed separately in each cohort.

### RNA extraction and lncRNA expression analysis

Total RNA was purified from frozen tissue with TriZol® Reagent (Life Technologies) as per manufacturer’s specifications. cDNA was synthetized from 500 ng of total RNA with The High Capacity cDNA Reverse Transcription Kit® (Applied Biosystems). TaqMan assays (Life Technologies) were used to quantify *HOTTIP* (Hs00955374_s1) in a 7500 Real Time PCR device (Applied Biosystems). *CDKN1B* (Hs00153277_m1) was used as endogenous control, and the mean of the HOTTIP expression in the normal tissue was used as calibrator sample to apply the2^-ΔΔCt^ method.

### Statistics

R v3.3 and IBM SPSS Statistics 22 were used for statistical analysis. TTR was calculated as the time between surgery and relapse or last follow-up and OS as the time between surgery and death from any cause or last follow-up. Kaplan-Meier survival curves and log-rank test were used for the survival analysis. All clinic-pathological factors with *p*-value ≤0.1 in the univariate analysis were included in the Cox multivariate regression analyses. The Optimal cutoff for the analysis of the impact on survival of the HOTTIP expression was identified using the Maxstat package (R Statistical Package) and validated by the Kaplan-Meier test. Paired (when necessary) or non-paired t-tests were used for comparisons between two groups. The independent validation of the prognostic role of HOTTIP levels were performed using TANRIC [28] **(**http://ibl.mdanderson.org/tanric/_design/basic/index.html**)** based upon data generated by the TCGA Research Network (http://cancergenome.nih.gov/).

## Results

### Patients

The main clinic-pathological characteristics of the patients are summarized in Table 1. Briefly, the mean patient age was 66 years (range, 32–84), 77 patients (77.8%) were male, 20 (20.2%) had Eastern Cooperative Oncology Group (ECOG) performance status (PS) 0 and 79 (79.8%) had PS 1. Seventy-three (60.2%) patients were diagnosed in stage I disease. Adenocarcinoma histology was found in fifty-five (55.5%) patients and 87 (87.9%) were active or former smokers. Median follow-up time was 44 months (range, 8–98). After a follow-up of 98 months, 31 (31.3%) patients experienced disease recurrence. Twenty-three patients (23.2%) received adjuvant therapy after surgery (17 stage II and six stage IB). Thirty-three percent of the patients harbored TP53 mutations and 20% harbored KRAS mutations. Adenocarcinoma patients with EGFR mutations (29.8%) showed a trend towards shorter TTR (*p* = 0.09) and OS (*p* = 0.09).

**Table 1:** Main clinical characteristics of the patients

Characteristics	Value	*N* = 99 N (%)
Sex	Male	77 (77.8)
	Female	22 (22.2)
Age, yrs.	Mean (Range)	66 (32–84)
	<=65	49 (49.5)
	> 65	50 (50.5)
ECOG PS^a^	0	20 (20.2)
	1	79 (79.8)
Stage	I	73 (73.7)
	II	26 (26.3)
T	1	47 (47.5)
	2	49 (49.5)
	3	3 (3)
N	0	88 (88.9)
	1	11 (11.1)
Histology	Adenocarcinoma	55 (55.5)
	Squamous cell carcinoma	36 (36.4)
	Others	8 (8.1)
Type of surgery	Lobectomy/bilobectomy	89 (89.9)
	Pneumonectomy	4 (4)
	Atypical Resection^b^	6 (6.1)
Smoking history	Current Smoker	34 (34.4)
	Former Smoker	53 (53.5)
	Never smoker	12 (12.1)
Adjuvant chemotherapy	Yes	23 (23.2)
	No	76 (76.8)
Relapse	No	68 (68.7)
	Yes	31 (31.3)
Emphysema	Yes	48 (48.5)
	No	44 (44.4)
	Unknown	7 (7.1)
COPD^c^	Yes	64 (64.6)
	No	35 (35.4)
DLCO^d^ (%)	Mean (Range)	73.4 (35–101)
Molecular features	TP53 mutations	31/94 (33)
	KRAS mutations	20/98 (20.4)
	EGFR mutations^e^	14/47 (29.8)

^a^ECOG PS, Eastern Cooperative Oncology Group performance status

^b^Atypical resection refers to Wedge resection, a non-anatomic limited resection of a lung portion

^c^COPD, chronic obstructive pulmonary disease

^d^DLCO, diffusing capacity of the lung for carbon monoxide

^e^EGFR mutational status was assessed only in adenocarcinoma patients

### HOTTIP expression and clinical characteristics

**Fig. 1:**
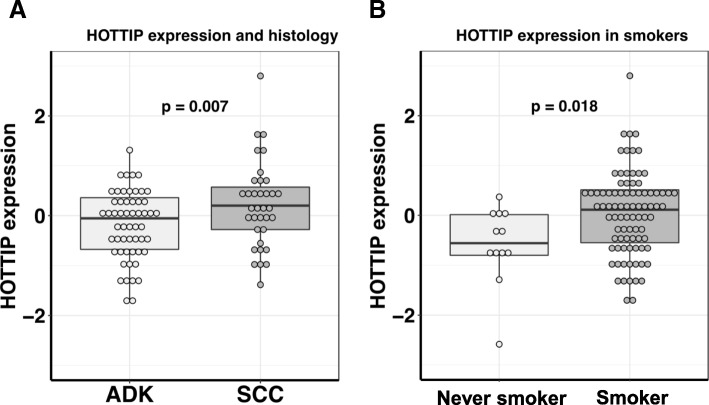
HOTTIP expression according to (**a**) histology and (**b**) smoking status. ADK, adenocarcinoma; SCC, squamous cell carcinoma

Patients with squamous cell carcinoma had significantly higher levels of *HOTTIP* than those with adenocarcinoma (*p* = 0.007; Fig. 1a). *HOTTIP* was also overexpressed in current and former smokers in comparison with never-smokers (*p* = 0.02; Fig. 1b) and in former smokers of < 15 years compared to former smokers of > 15 years although this difference was not significant (data not shown). Patients with PS 1 had higher levels of *HOTTIP* than those with PS 0 (*p* = 0.08). No association was observed between *HOTTIP* levels and TP53, KRAS or EGFR mutational status.

### HOTTIP expression and clinical outcome

**Fig. 2:**
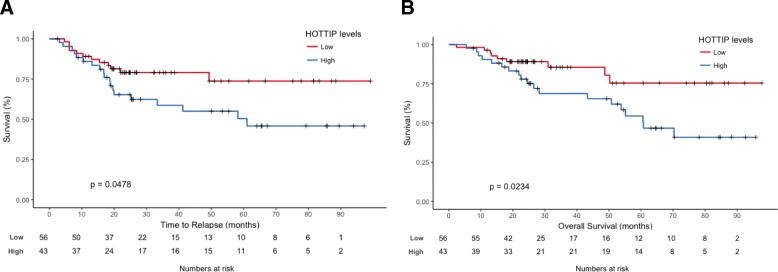
**a** Time to relapse and (**b**) overall survival according to HOTTIP levels

*HOTTIP* was expressed in all samples. Using the cutoff identified by the Maxstat package of R, patients were included depending on HOTTIP expression level into the high (*n* = 43) or the low (*n* = 56) group. The cutoff identified coincided with the Mean + SD of HOTTIP expression in the normal tissue (Additional file 1: Figure S1). Patients with high levels of *HOTTIP* had shorter TTR (78.3 vs 58 months; *p* = 0.05) and shorter OS (81.2 vs 61 months; *p* = 0.02) than those with low levels (Fig. 2).

The univariate Cox analysis for all clinical variables and for *HOTTIP* levels are shown in Table 2. In the multivariate analysis for TTR, including sex and *HOTTIP* levels, only *HOTTIP* levels emerged as a significant marker (Hazard ratio [HR]: 2.05; 95% confidence interval [CI]: 1–4.22; *p* = 0.05). The multivariate analysis for OS, including sex, age, PS, smoking status, emphysema, and *HOTTIP* levels, identified age > 65 (HR: 2.72; 95%CI: 1.04–5.13; *p* = 0.01) and *HOTTIP* levels (HR: 2.31, 95%CI: 1.04–5.13; *p* = 0.04) as independent prognostic markers (Table 3).

**Table 2:** Cox univariate analyses of time to relapse and overall survival

	Hazard Ratio (95%CI)	*p*-value
Time to Relapse		
Male sex	3.039 (0.924–9.999)	0.067
Age > 65	1.207 (0.596–2.443)	0.602
PS 1	1.738 (0.607–4.972)	0.303
Stage II	0.888 (0.397–1.989)	0.773
T3	1.213 (0.159–9.275)	0.853
N1	1.142 (0.399–3.272)	0.805
Histology-Adenocarcinoma	0.860 (0.402–1.840)	0.698
Type of surgery-Pneumonectomy	4.812 (0.494–46.847)	0.176
Smoker	1.295 (0.632–2.656)	0.480
Adjuvant chemotherapy-Yes	0.890 (0.382–2.072)	0.787
Emphysema	1.121 (0.540–2.329)	0.759
COPD	1.131 (0.532–2.403)	0.749
TP53 mutated	1.841 (0.888–3.814)	0.101
KRAS mutated	1.217 (0.544–2.721)	0.633
EGFR mutated	2.334 (0.845–6.450)	0.102
High HOTTIP levels	2.047 (0.992–4.224)	0.053
Overall Survival		
Male sex	3.748 (0.889–15.8)	0.072
Age > 65	2.628 (1.185–5.829)	0.017
PS 1	6.726 (0.914–49.520)	0.061
Stage II	0.720 (0.291–1.781)	0.478
T3	1.486 (0.192–11.507)	0.705
N1	0.585 (0.139–2.469)	0.466
Histology Adenocarcinoma	0.763 (0.336–1.732)	0.518
Type of surgery- Pneumonectomy	1.515 (0.213–10.797)	0.678
Smoker	0.387 (0.156–0.959)	0.040
Adjuvant chemotherapy- Yes	1.082 (0.459–2.549)	0.857
Emphysema	1.948 (0.897–4.230)	0.092
COPD	1.115 (0.504–2.467)	0.788
TP53 mutated	1.271 (0.568–2.844)	0.560
KRAS mutated	1.124 (0.477–2.646)	0.789
EGFR mutated	2.551 (0.822–7.916)	0.105
High HOTTIP levels	2.442 (1.1–5.418)	0.028

**Table 3:** Multivariate analyses of time to relapse and overall survival

	Hazard Ratio (95% CI)	*p*-value
Time to Relapse		
Male sex	2.71 (0.82–8.99)	0.10
High HOTTIP expression	2.05 (1–4.22)	0.05
Overall Survival		
Male sex	3.25 (0.76–13.84)	0.11
Age > 65	2.72 (1.23–6.04)	0.01
ECOG PS 1^a^	4.04 (0.54–30.17)	0.17
Smoker	0.60 (0.06–5.3)	0.66
Emphysema	1.5 (0.68–3.34)	0.32
High HOTTIP expression	2.31 (1.04–5.13)	0.04

^a^ECOG PS, Eastern Cooperative Oncology Group performance status

### Independent validation of HOTTIP prognostic impact using TCGA data

**Fig. 3:**
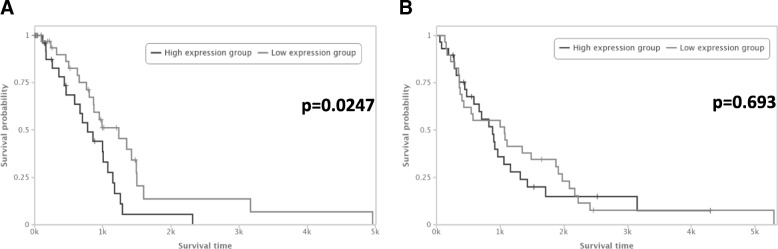
Independent validation of HOTTIP as prognostic marker in (**a**) TCGA lung adenocarcinoma (LUAD) patients and (**b**) TCGA squamous cell carcinoma (LUSC) patients

Using TCGA data and the TANRIC web tool, we found that high levels of *HOTTIP* were associated with shorter OS (*p* = 0.025) in a cohort of 91 stage I-II patients with lung adenocarcinoma (Fig. 3a). However, the same analysis in the TCGA cohort of 59 stage I-II patients with squamous cell carcinoma of the lung showed no significant differences (*p* = 0.69, Fig. 3b).

### Analysis of HOTTIP correlation with TCGA mRNA and miRNA data

**Fig. 4:**
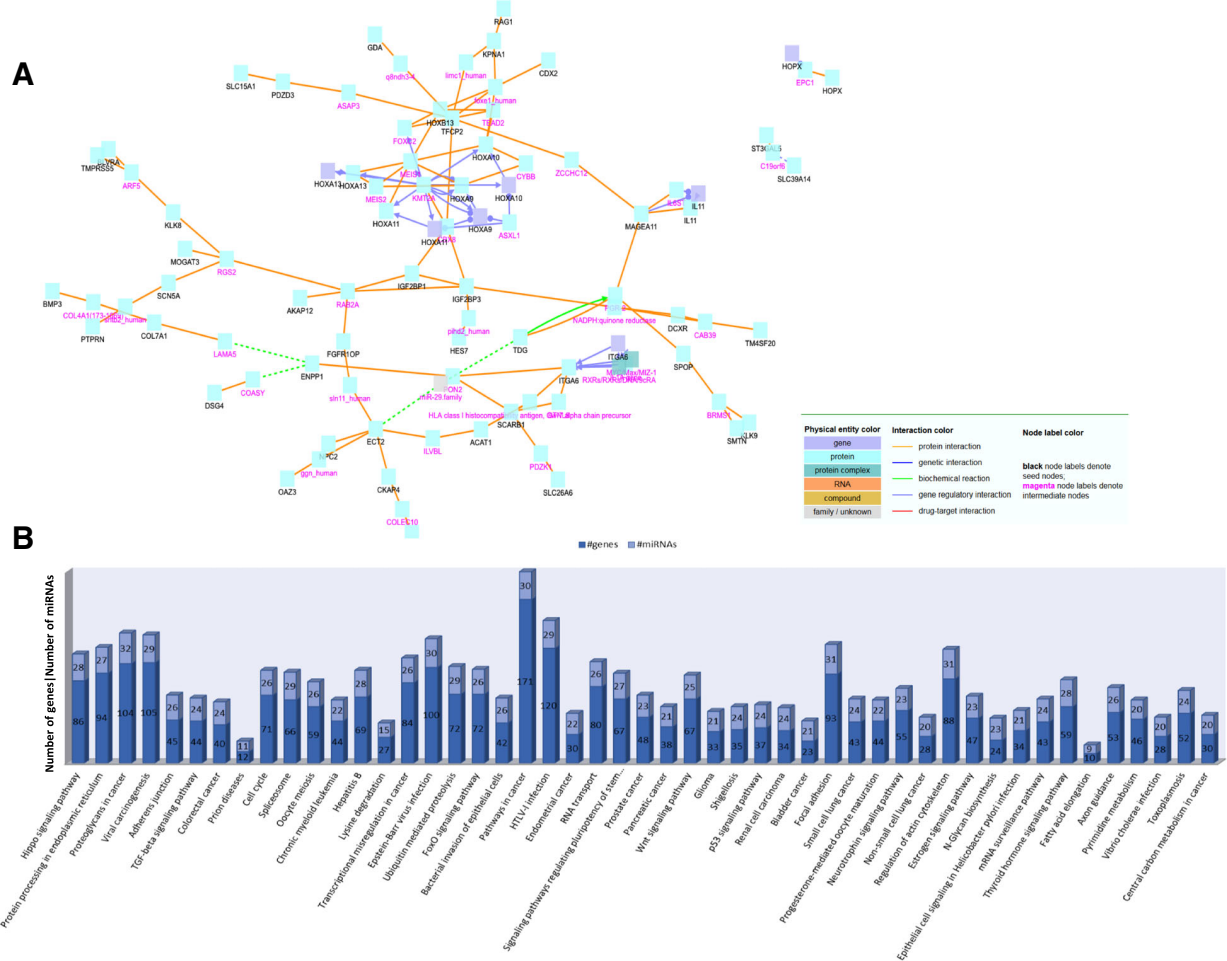
**a** Network analysis using the top 100 genes whose expression correlates with HOTTIP levels. The Induced Network Module Analysis tool from Consensus PathDB was used. **b** DIANA-miRPath analysis using the miRNAs whose expression correlates with HOTTIP levels. DIANA-mirPath is a miRNA pathway analysis web-server that examines a list of miRNAs provided by the user, identifies their potential/validated targets, and performs a pathway analysis to identify the most relevant pathways regulated by the miRNAs. In the present study, experimentally validated miRNA interactions derived from DIANA-Tarbase and KEGG analysis were used in the analysis. The KEGG signaling pathways identified are ordered from left to right in descending order of *p*-value. The y-axis indicates the number of genes (dark blue) and miRNAs (light blue) identified in the miRNA/target interaction analysis which are included in each KEGG pathway identified. For example, the Hippo signaling pathway is the most significant pathway identified and included 28 miRNAs from the HOTTIP-related miRNA signature, which targets 86 genes included in the Hippo signaling pathway

Using TANRIC and TCGA lung adenocarcinoma patients, we identified 1203 mRNAs and 61 miRNAs that significantly correlated with *HOTTIP* expression (*p* < 0.05). The most significant mRNA was *HOXA13* (r = 0.7, *p* < 0.001). Moreover, other *HOXA* genes were among the top 100 mRNAs, including *HOXA9* (r = 0.51, *p* < 0.001), *HOXA11* (r = 0.48, *p* < 0.001), and *HOXA10* (r = 0.44, *p* < 0.001). The Induced Network Module Analysis tool from Consensus PathDB (http://cpdb.molgen.mpg.de/CPDB) showed that most of the genes whose expression correlated with *HOTTIP* were closely related to the *HOX* gene network (Fig. 4a). One of the most significant miRNAs identified was miR-196b (r = 0.44, *p* < 0.001), which is the only miRNA located in the *HOXA* genomic region. We then used miR-Path v.3 [29] to study the putative pathways regulated by this *HOTTIP*-miRNA signature and identified 49 KEGG pathways (*p* < 0.05). The most significant pathway identified was the Hippo signaling pathway (*p* < 0.001), where 86 genes are Tarbase-validated targets of 28 of the miRNAs included in the signature. Other relevant KEGG pathways identified were the TGF-beta signaling pathway, Cell cycle, Signaling pathways regulating pluripotency of stem cells, Wnt signaling pathway and p53 signaling pathway (Fig. 4b). Additional file 2 includes the list of the top 100 mRNAs and all 61 miRNAs identified.

## Discussion

*HOTTIP* is one of the lncRNAs located in the *HOXA* genomic region of chromosome 7. The *HOX* genes are crucial transcription factors that determine the identity of cells and tissues during embryogenesis [30]. In adult tissues, *HOX* genes play a role in normal hematopoiesis regulation and are overexpressed in hematological [31] and solid cancers [10], including NSCLC [14]. Specifically, the *HOXA* gene cluster, where *HOTTIP* is found, plays a critical role in the patterning of tissues with mesodermal components, such as the lung, and in the regulation of epithelial–mesenchymal interactions [32]. *HOTTIP* has been related to tumor metastasis through induction of epithelial-mesenchymal transition [33].

In the present study, we have examined the role of *HOTTIP* as a prognostic factor in early-stage NSCLC patients treated with curative surgery and found that patients with higher levels of *HOTTIP* had shorter TTR and shorter OS than patients with low levels. Moreover, *HOTTIP* emerged as an independent prognostic factor in the multivariate analysis. The prognostic role of *HOTTIP* levels has been described in several cancers [19, 20, 22–25] and analyzed in several meta-analyses [26, 27, 34–36], which concluded that high *HOTTIP* expression in cancer patients is associated with poor clinical outcome. However, to the best of our knowledge, ours is the first study to provide evidence that *HOTTIP* impacts prognosis in NSCLC.

Additionally, we validated our findings on the prognostic value of *HOTTIP* levels in another patient population using TGCA data and the TANRIC webtool [28], showing that *HOTTIP* impacts prognosis in NSCLC patients with adenocarcinoma but not in those with squamous cell carcinoma. In our cohort, tumor *HOTTIP* levels were significantly overexpressed in squamous cell carcinoma compared to adenocarcinoma. While this may suggest that the prognostic impact of *HOTTIP* could differ between the main histological subtypes, the sub-analysis in the different histological subtypes in our cohort (Additional file 3: Figure S2) did not produce conclusive results, probably due to the small size of the sub-groups (55 adenocarcinomas and 36 squamous cell carcinomas). However, in both cases, high HOTTIP levels were associated with shorter TTR and shorter OS. Previous studies have reported that disordered patterns of HOX gene expression – specifically, *HOXA1*, *HOXA5* and *HOXA10* – are involved not only in the development of NSCLC but also in histological diversity [13]. *HOTTIP* is located close to the 3′ region of *HOXA10* and we observed a positive correlation in the expression of the two genes in the in silico analysis of TCGA data.

We also observed a significant upregulation of *HOTTIP* in current and former smokers in comparison with never smokers. An in vivo study showed that cigarette smoke increases mRNA and protein levels of *HOXA* in endometrial cells [37]. Interestingly, in our cohort, former smokers of > 15 years showed lower levels of *HOTTIP* than former smokers of < 15 years although this difference was not significant.

Finally, since regulatory interactions between lncRNAs, mRNAs and miRNAs have been described [38], we explored a possible association between the mRNAs and miRNAs whose expression correlated with *HOTTIP* according to TGCA data on adenocarcinoma NSCLC analyzed with TANRIC. This analysis identified a signature of 1203 mRNAs and 61 miRNAs. When we focused on the top 100 mRNAs, we observed that most of the genes whose mRNA expression correlated with *HOTTIP* expression were closely related to the HOX gene network, including *HOXA9, HOXA10, HOXA11, HOXA13*, and other *HOX* cluster members, such as *HOXB13*. Several studies have reported that interactions between *HOTTIP* and some of these *HOX* genes promote tumorigenesis. In prostate cancer, *HOTTIP* forms a complex with the transcription factor *TWIST1* and with *WDR5* and produces upregulation of *HOXA9* levels through chromatin regulation which correlates with an aggressive cellular phenotype [39]. Moreover, *HOTTIP* modulates cancer stem cell properties by binding *WDR5* and activating *HOXA9*, which enhances the Wnt/β-catenin pathway in prostate cancer stem cells [40]. In pancreatic cancer cells, *HOTTIP* regulates *HOXA10*, *HOXB2*, *HOXA11*, *HOXA9* and *HOXA1*, but not *HOXA13* [41]. However, *HOTTIP* and *HOXA13* have been associated with disease progression and worse outcome in hepatocellular carcinoma [22], with progression and gemcitabine resistance in pancreatic cancer [42], and with tumorogenesis and metastasis in esophageal squamous carcinoma [43] and gastric cancer [44]. In line with our results in NSCLC patient samples, these studies have shown that *HOTTIP* upregulation is associated with increased levels of *HOXA13*. In contrast, however, an in vitro study in the A549 NSCLC cell line showed that silencing *HOTTIP* led to increased *HOXA13* levels [21]. Although this study included only one cell line and did not analyze the correlation between *HOXA13* and *HOTTIP* in patient samples, it showed that *HOTTIP* acts as an oncogene, regulating apoptosis, proliferation, and migration in NSCLC. Another study, by Zhang et al., also reported the role of HOTTIP as oncogene in A549 through regulation of the AKT signaling pathway. The authors showed that the overexpression of HOTTIP enhanced proliferation and paclitaxel resistance [45].

Further studies are needed to clarify this interaction, including analyses in other NSCLC cell lines. Interestingly, the network analysis also identified an additional node that included *HOXP*, a transcription factor related to alveolar differentiation, whose suppression has been linked to increased invasiveness in adenocarcinoma lung cancer [46]. There was a negative correlation between *HOXP* and *HOTTIP* levels, which could explain the more aggressive phenotype we have observed in patients with high *HOTTIP* levels.

When we explored the miRNAs in the 61-miRNA signature identified in the TANRIC analysis, we found a positive correlation with miR-196b, located in the distal part of the same *HOXA* cluster, and with miR-196a-1, located in the *HOXB* cluster. The study of the potential pathways regulated by the miRNA signature showed the importance of the *Hippo* signaling pathway, which has previously been shown to be altered in NSCLC [47].

## Conclusions

Our findings provide the first indication that *HOTTIP* may be a prognostic biomarker in NSCLC. In line with the prognostic impact of *HOTTIP* levels in other cancers, high levels of *HOTTIP* correlated with worse TTR and worse OS in our early-stage NSCLC patients. *HOTTIP* may be useful for the identification of resected NSCLC patients at high risk of relapse. Further investigation in a prospective study is warranted to validate these findings and to examine potential *HOTTIP*-based therapeutic approaches.

## Additional files

Additional file 1: **Figure S1.** (A) Time to relapse and (B) overall survival according to HOTTIP levels in adenocarcinoma patients. (C) Time to relapse and (D) overall survival according to HOTTIP levels in squamous cell carcinoma patients. (PPTX 57 kb)

Additional file 2: Excel file with the results of the analysis of HOTTIP correlation with TCGA data that includes 2 sheets: mRNAs sheet, which includes the list of the top 100 mRNAs; and miRNAs sheet with all 61 miRNAs identified. (XLSX 19 kb)

Additional file 3: **Figure S2.** Bar plot showing the 99 patients ordered by HOTTIP expression level. An arrow shows the mean HOTTIP expression in the normal tissue and the cutoff used to classify the patients in high or low expression. The cutoff coincides with the Mean + SD of HOTTIP expression in the normal tissue. (PPTX 104 kb)

## Abbrebiations

ECOG: Eastern Cooperative Oncology Group
lncRNAs: long non-coding RNAs
miRNAs: microRNAs
ncRNAs: non-coding RNAs
NSCLC: Non-small-cell lung cancer
OR: Odds ratio
OS: Overall survival
PS: Performance status
TCGA: The Cancer Genome Atlas
TTR: Time to relapse
