# Supplementary figures and images for: Clinical significance of long non-coding RNA HOTTIP in early-stage non-small-cell lung cancer
Source: BMC Pulm Med. 2019 Feb 28;19:55. doi: 10.1186/s12890-019-0816-8 (PMC6393998; doi:10.1186/s12890-019-0816-8)

## Slide 1
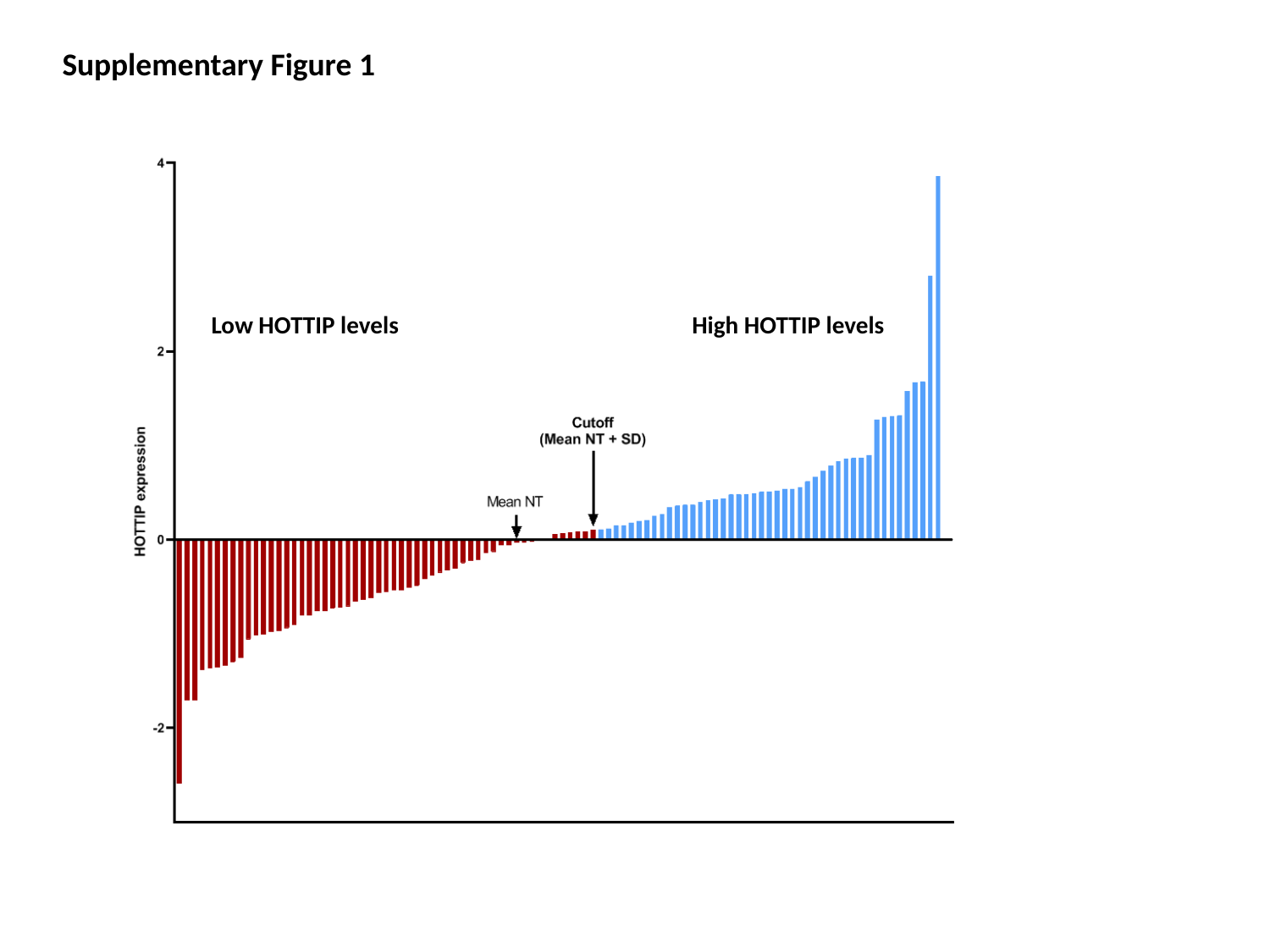

Supplementary Figure 1
Low HOTTIP levels
High HOTTIP levels

Supplement: Supplementary file 1 — Figure S1. (A) Time to relapse and (B) overall survival according to HOTTIP levels in adenocarcinoma patients. (C) Time to relapse and (D) overall survival according to HOTTIP levels in squamous cell carcinoma patients. (PPTX 57 kb) [file 12890_2019_816_MOESM1_ESM.pptx]

## Slide 1
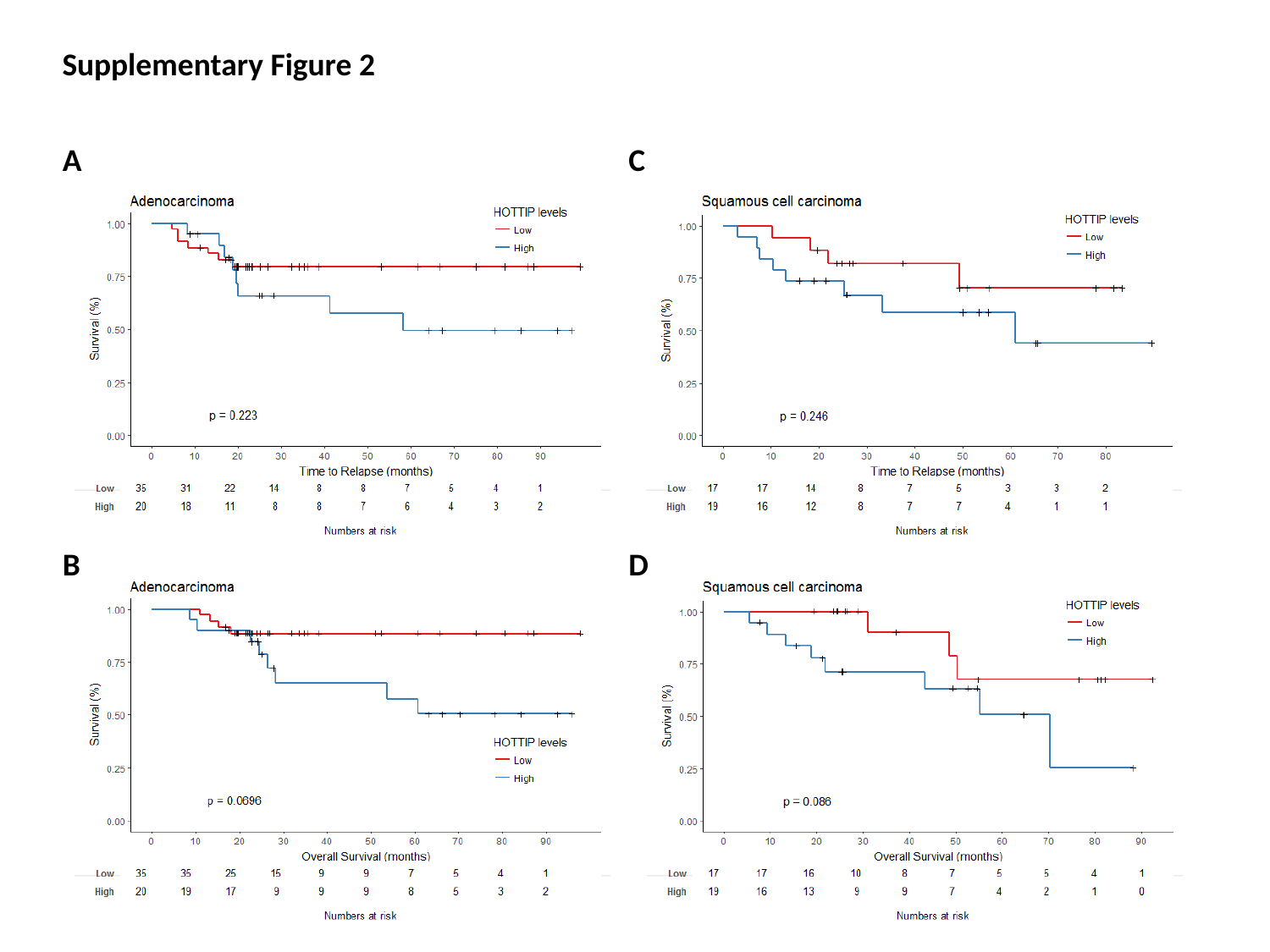

Supplementary Figure 2
A
C
B
D

Supplement: Supplementary file 3 — Figure S2. Bar plot showing the 99 patients ordered by HOTTIP expression level. An arrow shows the mean HOTTIP expression in the normal tissue and the cutoff used to classify the patients in high or low expression. The cutoff coincides with the Mean + SD of HOTTIP expression in the normal tissue. (PPTX 104 kb) [file 12890_2019_816_MOESM3_ESM.pptx]
